# Most microsatellite unstable sporadic colorectal carcinomas carry *MBD4* mutations

**DOI:** 10.1054/bjoc.2000.1482

**Published:** 2000-12

**Authors:** S Bader, M Walker, D Harrison

**Affiliations:** Sir Alastair Currie C.R.C. Laboratories, Department of Pathology, Molecular Medicine Centre, University of Edinburgh, Crewe Road, Edinburgh, EH4 2XU, UK

**Keywords:** MBD4, colorectal cancer, microsatellite instability

## Abstract

The *MBD4* gene is involved in the repair of mutation at methyl-CpG dinucleotides. In microsatellite unstable tumours *MBD4* can itself be mutated at an exonic polynucleotide tract. By analysing DNA from microdissected tumour samples we have found that both frequency and pattern of mutation are more significant than originally reported. © 2000 Cancer Research Campaign http://www.bjcancer.com


*British Journal of Cancer* (2000) **83**(12), 1646–1649
© 2000 Cancer Research Campaign
doi: 10.1054/ bjoc.2000.1482, available online at http://www.idealibrary.com on IDEAL®

Short Communication

### Most microsatellite unstable sporadic colorectal carcinomas carry *MBD4* mutations

**S Bader, M Walker and D Harrison**

Sir Alastair Currie C.R.C. Laboratories, Department of Pathology, Molecular Medicine Centre, University of Edinburgh, Crewe Road, Edinburgh EH4 2XU, UK

**Summary** The *MBD4* gene is involved in the repair of mutation at methyl-CpG dinucleotides. In microsatellite unstable tumours *MBD4* can itself be mutated at an exonic polynucleotide tract. By analysing DNA from microdissected tumour samples we have found that both frequency and pattern of mutation are more significant than originally reported. © 2000 Cancer Research Campaign http://www.bjcancer.com

**Keywords:** MBD4; colorectal cancer; microsatellite instability

The *MBD4* gene encodes a thymine glycosylase that binds to DNA at methyl-CpG dinucleotide sites and can excise the thymine base at a T.G mismatched basepair resulting from deamination of the methyl-C (Hendrich and Bird, 1998; Bellacosa et al, 1999; Hendrich et al, 1999). The gene is therefore thought to be an important component of the system that maintains the integrity of methylation patterns throughout the genome, and so silencing of gene transcription. *MBD4* contains a polynucleotide stretch of 10 adenines ($A_{10}$) within one translated exon that we and others have found to be mutated in at least 25% of mismatch repair defective (RER+) colorectal tumours (Bader et al, 1999; Riccio et al, 1999). The mutation involves either gain or loss of one base and in either case is predicted to cause a premature cessation of translation and production of non-functional protein. It has been suggested from in vitro studies (Bellacosa et al, 1999) that *MBD4* may play a role in the cause of microsatellite instability but this would not be borne out by our observations of mutations seen in vivo for several reasons. Firstly, mutations within the $A_{10}$ tract would generate a truncated protein that has lost domains including that shown to interact with MLH1. Secondly, the apparently relatively low frequency of mutation and the strict association of mutation with the RER+ phenotype strongly suggests that *MBD4* abnormalities are actually downstream from mismatch repair (MMR) gene defects. Here we report more accurately the frequency and timing of *MBD4* mutation in established RER+ sporadic colorectal carcinomas by microdissecting multiple and independent formalin-fixed, paraffin-embedded tumour sections to ensure less than 5% stromal contamination.

### MATERIALS AND METHODS

Patients studied were all of those used before in Bader et al (1999) which were microsatellite unstable. 10 μm sections were cut from 10% formalin-fixed paraffin-embedded tissues, dewaxed and stained with eosin. These were aligned with haematoxylin & eosin stained contiguous sections previously scrutinized and marked to identify tumour-rich areas. Selected areas were covered with 10 μl deionized water, scraped with a fresh scalpel blade and transferred into a sterile microfuge tube. The tissue suspension was digested with proteinase K overnight at 42˚C, then extracted once with phenol and chloroform, ethanol precipitated and resuspended in deionized water. Each microdissected site was derived from a section made from an independent paraffin-embedded tumour block, and so represented a region from the same tumour but physically separated from other microdissected sites. PCR amplification and SSCP analyses were done as described in Bader et al (1999).

### RESULTS

Mutations were identified in many of the microdissected sites taken from the same patients as originally assayed from frozen tumour samples. We detected mutations in at least one site in 17 of 19 (89%) primary tumours (see Table 1). Some of these, for example case 7 sites b and c, show reduction to homozygosity. In three cases (8, 9 and 15) we found no mutations in microdissected sites despite a mutation in the frozen sample. This was probably a chance occurrence due to different sites of sampling picking up the heterogeneity present in the tumour of these patients. This is easily true for cases 9 and 15 where only three sites were assayed. Figure 1 shows the results for three example cases where whole tissue tumour sample originally gave a negative (apparent lack of mutation) result. Within each tumour there was considerable heterogeneity, implying that the point during clonal development of each tumour when the *MBD4* gene sustains mutation may occur late after tumorigenesis has occurred. Theoretically it is possible that a loss or gain of a base could be reverted due to the continuing microsatellite unstable environment, but this is unlikely. Clearly, *MBD4* is not mutated in the founder clone of any tumour, nor is it mutated at as early a stage as reported for transforming growth factor β type II receptor (TGFβRII). However 11 of the 17 (65%) cases positive for *MBD4* mutation had abnormalities in a majority of sites (more than 50% of all sites tested), suggesting that mutation can be relatively early.

*Received 11 May 2000*
*Revised 24 July 2000*
*Accepted 14 August 2000*

*Correspondence to:* S Bader

**Table 1** *MBD4* mutations of RER+ sporadic colorectal cancers.

| Case | Microdissected site in primary tumour | Frozen tissue data* | Microdissected data‡ | Dukes stage |
|---|---|---|---|---|
| c1 | | wt | | B |
| | a | | wt | |
| | b | | wt | |
| | c | | wt | |
| c2 | | wt | | B |
| | a | | wt/–1 | |
| | b | | –1 | |
| | c | | wt | |
| c3 | | wt | | B |
| | a | | wt/–1 | |
| | b | | wt/–1 | |
| | c | | wt/–1 | |
| c4 | | wt | | B |
| | a | | wt | |
| | b | | wt | |
| | c | | wt | |
| | d | | wt/–1 | |
| c5 | | wt | | A |
| | a | | wt | |
| | b | | wt | |
| | c | | wt/–1 | |
| c7 | | wt | | B |
| | a | | wt | |
| | b | | –1 | |
| | c | | –1 | |
| | d | | wt | |
| | e | | wt/–1 | |
| c8 | | wt/–1 | | B |
| | a | | wt | |
| | b | | wt | |
| | c | | wt | |
| | d | | wt | |
| | e | | wt | |
| c9 | | wt/–1 | | B |
| | a | | wt | |
| | b | | wt | |
| c10 | | wt/–1 | | B |
| | a | | wt | |
| | b | | wt/–1 | |
| c11 | | wt/–1 | | C |
| | a | | wt | |
| | b | | wt | |
| | c | | wt/–1 | |
| c13 | | wt | | B |
| | a | | wt | |
| | b | | wt | |
| | c | | wt | |
| | d | | wt | |
| | e | | wt | |
| | f | | wt/–1 | |
| | g | | wt | |
| | h | | wt | |
| c14 | | wt | | A |
| | a | | wt/–1 | |
| | b | | wt/–1 | |
| c15 | | wt/–1 | | A |
| | a | | wt | |
| | b | | wt | |
| c16 | | wt/–1 | | A |
| | a | | wt/–1 | |
| | b | | wt | |
| c17 | | wt | | C |
| | a | | wt/+1 | |
| | b | | –1 | |
| | c | | wt/+1 | |
| | d | | wt/–1/+1 | |

*© 2000 Cancer Research Campaign*

| | | | | |
|---|---|---|---|---|
| c18 | | wt/–1 | | C |
| | a | | wt/–1 | |
| | b | | –1 | |
| | c | | wt/–1 | |
| c19 | | wt | | B |
| | a | | –1 | |
| | b | | –1 | |
| | c | | –1 | |
| c20 | | wt | | C |
| | a | | wt | |
| | b | | wt | |
| c21 | | wt/–1 | | B |
| | a | | –1 | |
| | b | | –1 | |
| | c | | wt | |
| | d | | wt | |

* shows samples assayed by cycle sequencing or end labelled non-denaturing gel electrophoresis; ‡ shows samples assayed by end labelled non-denaturing gel electrophoresis. 'wt' or '–1' indicates homo/hemizygous, wild type or –1 adenine respectively; 'wt/–1' or 'wt/+1' indicates heterozygous wild type and –1 or +1 adenine respectively.

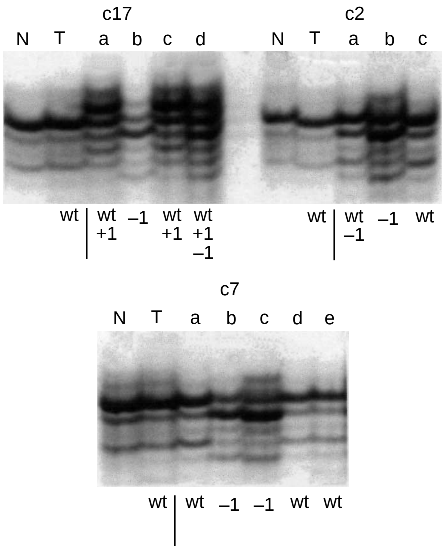

**Figure 1** Mutation of *MBD4* in microdissected sites of three example cases. 'N' indicates a sample of frozen whole normal colon mucosa; 'T' indicates a sample of frozen whole colorectal tumour; 'a–e' represent independent sites microdissected from paraffin sections.

### DISCUSSION

We have found, upon analysis of microdissected sites rather than total tumour tissue, that *MBD4* mutations occur in 89% of colorectal cancers. This is significantly more frequent than in previous reports (43% in Bader et al (1999), 25% in Riccio et al (1999)). There is, however, a wide range in proportion of mutation positive tumour areas within any one case. Such intratumoral heterogeneity of exonic polynucleotide tracts within sporadic colorectal and other cancers has been documented recently (Abdel-Rahman et al, 1999; Chung et al, 1999; Samowitz and Slattery, 1999), and implies a late occurrence of mutations in a gene during the progression of the tumour. Thus, *MBD4* is not mutated as frequently or as early in tumorigenesis as is the *TGFβRII* gene (Markowitz et al, 1995), but mutation is not as rare as for *IGFIIR* gene (Souza et al, 1996).

It is possible that the observed pattern of *MBD4* mutation merely reflects a non-functional consequence of microsatellite instability at this locus. However, mutations in *MBD4* downstream of MMR gene defects could still be important such that *MBD4* lack of repair of T.G mismatches compounds the MMR defects. The additional mutation load could either promote progression along the tumorigenic pathway, or may help to explain why many RER+ tumours have a better stage-specific prognosis (Bubb et al, 1996; Sankila et al, 1996). The suggestion that *MBD4* could be a tumour suppressor gene remains valid regarding biallelic inactivation. We found in this study frequent reduction to homozygosity, or the presence of +1 and –1 adenine biallelic mutation (for example Figure 1, case 17), and loss of heterozygosity of neighbouring polymorphic markers has also been reported (Riccio et al, 1999). The question thus remains, regarding the nature of the proposed biological significance of truncated forms of MBD4 protein at late stages of tumour development. We have hypothesized a dominant negative or gain of function phenotype in cells that are even only heterozygous for the frameshift mutations (Bader et al, 1999) and experiments to test this hypothesis are in progress. Hendrich and Bird (1998) have already shown in vitro that C-terminal deleted forms of MBD4 can still efficiently bind to methyl-CpG.

In summary, mutations occur in *MBD4* at higher frequencies than originally observed and to a significant degree as biallelic inactivation. It has been previously concluded that *MBD4* alterations occur as a downstream event from MMR gene mutation but the results presented here suggest that clonal mutations of *MBD4* may nevertheless play a significant role in disease evolution. These observations, together with the proposed function of the protein, suggest intriguingly that *MBD4* is important in determining the phenotype of RER+ sporadic colorectal tumours, particularly the later phases of malignancy.

### ACKNOWLEDGEMENTS

This work was supported in part by the Chief Scientist Office within the Scottish Executive (MW), and the Cancer Research Campaign (SB, DH).

### REFERENCES

Abdel-Rahman WM, Georgiades IB, Curtis LJ, Arends MJ and Wyllie AH (1999) Role of BAX mutations in mismatch repair-deficient colorectal carcinogenesis. *Oncogene* **18**: 2139–2142

 *© 2000 Cancer Research Campaign*

Bader S, Walker M, Hendrich B, Bird A, Bird C, Hooper M and Wyllie A (1999) Somatic frameshift mutations in the MBD4 gene of sporadic colon cancers with mismatch repair deficiency. *Oncogene* **18**: 8044–8047

Bellacosa A, Cicchillitti L, Schepis F, Riccio A, Yeung AT, Matsumoto Y, Golemis EA, Genuardi M and Neri G (1999) MED1, a novel human methyl-CpG-binding endonuclease, interacts with DNA mismatch repair protein MLH1. *Proc Natl Acad Sci USA* **96**: 3969–3974

Bubb VJ, Curtis LJ, Cunningham C, Dunlop MG, Carothers AD, Morris RG, White S, Bird CC and Wyllie AH (1996) Microsatellite instability and the role of hMSH2 in sporadic colorectal cancer. *Oncogene* **12**: 2641–2649

Chung Y-J, Kim K-M, Choi J-R, Choi S-W and Rhyu M-G (1999) Relationship between intratumoral histological heterogeneity and genetic abnormalities in gastric carcinoma with microsatellite instability. *Int J Cancer* **82**: 782–788

Hendrich B, Bird A (1998) Identification and characterisation of a family of mammalian methyl-CpG binding proteins. *Mol Cell Biol* 18: 6538–6547

Hendrich B, Hardeland U, Ng H-H, Jiricny J and Bird A (1999) The thymine glycosylase MBD4 can bind to the product of deamination at methylated CpG sites. *Nature* **401**: 301–304

Markowitz S, Wang J, Myeroff L, Parsons R, Sun LZ, Lutterbaugh J, Fan RS, Zborowska E, Kinzler KW, Vogelstein B, Brattain M and Willson JKV (1995) Inactivation of the Type II TGFβ receptor in colon cancer cells with microsatellite instability. *Science* **268**: 1336–1338

Riccio A, Aaltonen LA, Godwin AK, Loukola A, Percesepe A, Salovaara R, Masciullo V, Genuardi M, Paravatou-Petsotas M, Bassi DE, Ruggeri BA, Klein-Szanto AJP, Testa JR, Neri G and Bellacosa A (1999) The DNA repair gene MBD4 (MED1) is mutated in human carcinomas with microsatellite instability. *Nature Genet* **23**: 266–268

Samowitz WS and Slattery ML (1999) Regional reproducibility of microsatellite instability in sporadic colorectal cancer. *Genes Chromos & Cancer* **26**: 106–114

Sankila R, Aaltonen LA, Jarvinen HJ and Mecklin JP (1996) Better survival rates in patients with MLH1-associated hereditary colorectal cancer. *Gastroenterology* **110**: 682–687

Souza RF, Appel R, Yin J, Wang S, Smolinksi KN, Abraham JM, Zou T-T, Shi Y-Q, Lei J, Cottrel J, Cymes K, Biden K, Simms L, Leggett B, Lynch PM, Frazier M, Powell SM, Harpaz N, Sugimura H, Young J and Meltzer SJ (1996) Microsatellite instability in the insulin-like growth factor II receptor gene in gastrointestinal tumours. *Nature Genet* **14**: 255–257

*© 2000 Cancer Research Campaign*

